# Outbreak of Cyanide Poisoning Caused by Consumption of Cassava Flour — Kasese District, Uganda, September 2017

**DOI:** 10.15585/mmwr.mm6813a3

**Published:** 2019-04-05

**Authors:** Phoebe H. Alitubeera, Patricia Eyu, Benon Kwesiga, Alex R. Ario, Bao-Ping Zhu

**Affiliations:** ^1^Uganda Public Health Fellowship Program, Kampala, Uganda; ^2^CDC Uganda, Kampala, Uganda; ^3^Division of Global Health Protection, Center for Global Health, CDC.

Cassava (*Manihot esculenta*), an edible tuberous root that is resistant to drought, diseases, and pests, is a major source of carbohydrates in tropical areas, the second most widely grown and consumed food in Uganda after bananas, and a staple in the diet for approximately 57% of the Uganda population (Figure 1) (*1*). On September 5, 2017, a funeral was held in Kasese District in western Uganda. Following the funeral, 33 persons with symptoms that included diarrhea, vomiting, and abdominal pains were admitted to Bwera Hospital in Kasese District. On September 8, the Uganda Ministry of Health received notification from the Kasese District health team regarding this outbreak of suspected food poisoning. An investigation to determine the cause of the outbreak and recommend control measures revealed that the outbreak resulted from consumption of a cassava dish made by combining hot water with cassava flour. The implicated batch of cassava flour was traced back to a single wholesaler and found to contain high cyanogenic content. Informed by the investigation findings, police confiscated all cassava flour from retailers identified as the patients’ source of the flour. Health education about cyanide poisoning from cassava and the need to adequately process cassava to reduce cyanogenic content was conducted by public health officials.

**FIGURE 1:**
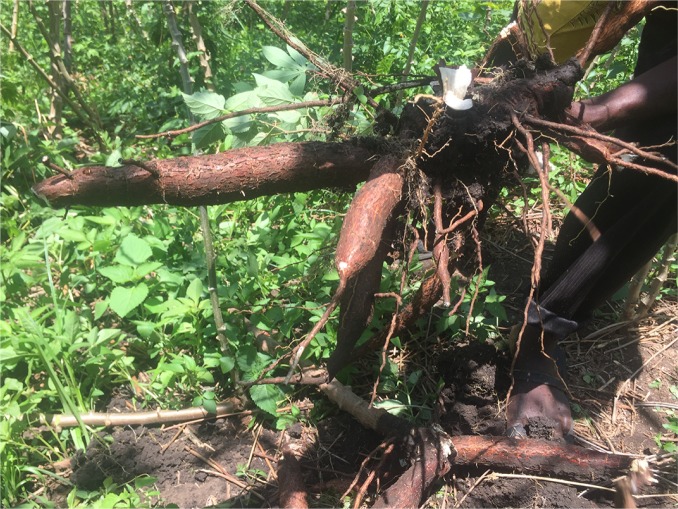
Approximately 600 million tropical residents, half of whom live in Africa, rely on cassava as their main food source Photo/Uganda Public Health Fellowship Program

## Epidemiologic Investigation

An investigation into the outbreak was conducted by fellows of the Uganda Public Health Fellowship Program and their supervisors. A probable case was defined as sudden onset of vomiting or diarrhea with one or more of the following signs or symptoms in a resident of one of three Kasese District subcounties during September 1–9, 2017: myalgia, tachycardia, tachypnea, headache, dizziness, lethargy, convulsions, or syncope. Medical records at Bwera Hospital, which has a catchment area covering the three subcounties, were systematically reviewed. Active case-searching was conducted with the help of community leaders.

The investigation identified 98 probable cases, with two deaths (case-fatality rate = 2%). The median patient age was 10 years (range = 11 months–75 years). Reported signs or symptoms included vomiting (95%), diarrhea (87%), malaise (60%), dizziness (48%), tachypnea (27%), syncope (16%), and tachycardia (10%); 6% of patients reported fever. These signs and symptoms suggested cyanide poisoning (*3*). Although the recommended treatment for acute cyanide toxicity is hydroxocobalamin (injectable vitamin B12) (*4*), persons who went to health care facilities were managed on intravenous antibiotics and oral rehydration salts.

**FIGURE 2:**
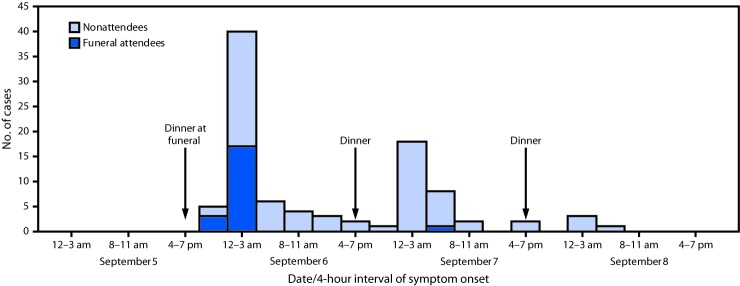
Number of cases of cyanide poisoning from eating a cassava flour dish, by date and 4-hour interval of symptom onset, among funeral attendees and nonattendees — Kasese District, Uganda, September 5–8, 2017

The outbreak affected all age groups; the attack rate was similar in males and females, and in all three subcounties, but was lower in persons aged 19–44 years (5.5 per 10,000 population) than in younger or older persons (≤18 years, 15.1 and ≥45 years, 12.1) (p = 0.003) (Table 1). Illness onset began a few hours after the funeral on September 5, and continued through September 8 (Figure 2). Among funeral attendees, a peak in cases occurred a few hours after the evening meal at the funeral; among nonattendees, three successively diminishing peaks occurred, each a few hours after the evening meals on September 6, 7, and 8 (Figure 2).

**TABLE 1:** Attack rates of cyanide poisoning, by age group, sex, and subcounty during an outbreak caused by eating a cassava flour dish that contained high cyanogenic content — Kasese District, Uganda, September 2017

Characteristic	No. of cases	Population*	Attack rate (per 10,000 population)
**Total**	98	84,032	11.7
**Age group (yrs)^†^**			
0–5	23	15,464	14.9
5–18	49	32,134	15.2
19–44	15	27,321	5.5
≥45	11	9112	12.1
**Sex^§^**			
Male	43	41,092	10.5
Female	55	42,940	12.8
**Subcounty^§^**			
Bwera	25	17,883	13.7
Ihandiro	19	13,881	14.0
Mpondwe Lubiriha Trading Centre	54	52,268	10.3

* Projected 2017 population based on the 2014 census.

^†^ Differences were statistically significant by Chi-square test (p = 0.003).

^§^ Differences were not statistically significant by Chi-square test (p>0.05).

A case-control study was conducted to identify the likely source of the outbreak. Two age-matched (within 5 years) controls for each case-patient were selected from among neighbors of case-patients who had eaten cassava during September 1–9 but did not develop vomiting or diarrhea. A total of 88 case-patients and 176 controls were interviewed in person regarding potential exposures. To account for the matched design, Mantel-Haenszel odds ratios (ORs) and the associated 95% confidence intervals (CIs) were computed, where the stratification variable was the match-set. Analyses were performed using CDC’s Epi Info software.

Case-patients were more likely than were controls to have attended the funeral (OR = 40; 95% CI = 5.4–298) and to have purchased their cassava flour from retailers that were supplied by wholesaler A (OR = infinity; 95% CI = 5.6–infinity) (Table 2). When the data were stratified by funeral attendance, all funeral attendees were noted to have eaten cassava purchased from a retailer supplied by wholesaler A. Among nonattendees, 100% of case-patients and 79.2% of controls bought cassava flour from retailers supplied by wholesaler A during the outbreak period (OR = infinity; Fisher’s exact 95% CI = 4.3–infinity).

**TABLE 2:** Exposure factors among case-patients and controls during a cyanide poisoning outbreak caused by eating a cassava flour dish that contained high cyanogenic content — Kasese District, Uganda, September 2017

Exposure factors	Case-patients (N = 88) No. (%)	Controls (N = 176) No. (%)	OR (95% CI)
**Attendance at September 5 funeral**			
Yes	21 (23.9)	3 (1.7)	40 (5.4–298*)
No	67 (76.1)	173 (98.3)	Referent
**Source of cassava during outbreak period**			
Ever purchased cassava from retailers supplied by wholesaler A	88 (100.0)	141 (80.1)	Infinity (5.4–infinity*)
Never purchased cassava from retailers supplied by wholesaler A	0 (0)	35 (19.9)	Referent
**Among funeral nonattendees^†,§^**			
Ever purchased cassava from retailers supplied by wholesaler A	67 (100.0)	137 (79.2)	Infinity (4.3–infinity^¶^)
Never purchased cassava from retailers supplied by wholesaler A	0 (0)	36 (20.8)	Referent

**Abbreviations**: CI = confidence interval; OR = odds ratio.

* Mantel-Haenszel OR and CI.

^†^ 67 case-patients and 173 controls.

^§^ Association between eating cassava from wholesaler A and illness could not be assessed among attendees at funeral because all funeral attendees ate cassava from wholesaler A.

^¶^ Fisher’s exact OR and CI.

## Traceback and Laboratory Investigations

The Uganda Public Health Fellowship Program investigators conducted interviews with area retailers and wholesalers regarding their sources of cassava, and the implicated product was further traced back to its source. Two primary sources were identified. Farmers grew their own cassava, known as “sweet” cultivars. Residents also bought cassava from retailers, especially for serving at communal gatherings when a large quantity was needed. The retailers bought their cassava flour from wholesalers, who mainly bought from cassava mills in Kasese town, approximately 31 miles (50 km) away. During the outbreak period, wholesaler A was the main supplier to retailers in the three subcounties. Wholesaler A reportedly bought the implicated batch from a town bordering Uganda and Tanzania, approximately 174 miles (280 km) from Kasese; the implicated batch was further traced back to Tanzania. Because this batch cost less than other batches for sale at the time, investigators speculated that it might have been from “wild” cultivars. This suspicion was corroborated by funeral attendees, who described the cassava flour dish served at the funeral as pure white, which is typical of flour from wild cultivars, instead of the creamy-colored flour from sweet cultivars.

Cassava flour samples were obtained for visual inspection and spectrophotometric cyanide testing by the Government Analytical Laboratory in Uganda. The five samples obtained from the implicated batch were pure white in color and contained cyanogenic glycoside that was equivalent to an average of 88 ppm of cyanide (range = 85–90), more than eight times the recommended safe level of 10 ppm (*2*).

Informed by findings of this investigation, police in Kasese District confiscated all sacks of cassava flour from retailers where affected families had purchased the product. Health education was conducted in the communities about cyanide poisoning from cassava and the need to adequately process cassava to reduce the cyanide content.

## Discussion

The epidemiologic, traceback, and laboratory investigations indicated that this outbreak of cyanide poisoning resulted from eating cassava with a high cyanogenic content. Patients’ signs and symptoms included dizziness, vomiting, tachypnea, syncope, and tachycardia and were consistent with acute cyanide poisoning (*3*,*5*); the absence of fever made infectious etiology unlikely. Symptoms occurred a few hours after meals during which a cassava flour dish was served. This finding was consistent with previous reports, with symptoms typically starting 4–6 hours after ingesting a meal, as the cyanide is released upon digestion of the cyanogenic glycosides (*6*). The case-control study strongly linked the outbreak to cassava flour supplied by wholesaler A, and the traceback investigation suggested that the implicated cassava might have originated in Tanzania. The laboratory investigation found high levels of cyanogenic glycosides in the implicated cassava flour.

Cassava crops are resistant to drought, pests, and diseases, making cassava invaluable for food security, especially in areas plagued by food shortages (*7*). Approximately 600 million tropical residents, half of whom live in Africa, rely on cassava as their main food source (*8*). Acute cyanide poisoning, often with fatal consequences, can occur after eating a large amount of cassava, especially in communities dependent on a monotonous cassava diet (*9*). Recurrent exposure to nonlethal concentrations through a monotonous cassava-based diet leads to long-term effects, including paralytic diseases such as tropical ataxic neuropathy and konzo, a neurologic disease characterized by sudden onset of irreversible, nonprogressive spastic paralysis (*2*). In sub-Saharan Africa, particularly Uganda, Tanzania, and the Democratic Republic of the Congo, thousands of persons might have experienced cyanide poisoning from cassava (*7*,*8*), but the full extent of the problem remains unknown because reliable data are lacking.

Although wild cassava cultivars have greater yield, higher resistance to pests, and longer storability in the soil than do sweet cultivars, they are bitter, and hence, have a lower market value. In addition, the cyanogenic content of wild cultivars is as high as 2,000 ppm of dry weight (*1*), 200 times the safe level (<10 ppm) recommended by the World Health Organization (*2*). Therefore, wild cultivars are not recommended for human consumption. However, some farmers still plant wild cultivars because of their resilience and high yield (*1*).

Although the cyanogenic content of sweet cassava is substantially less than that of wild cultivars (up to 100 ppm) (*1*), the sweet cassava cultivars still require detoxification before they are consumed; this involves peeling the tubers, soaking them in water for 4–6 days, and sun-drying or roasting them. The outer layer is then scraped off and the remainder ground into flour. This process promotes enzymatic degradation of cyanogenic glycosides. If the soaking or drying time is too short, enzymatic degradation will be inadequate, and cyanogenic glycosides remain high (*5*). During droughts, cassava traders sometimes fail to follow recommended procedures, which can result in a product with high levels of cyanogenic glycosides that can lead to cyanide poisoning (*1*).

A rapid, semiquantitative, colorimetric test that is free to workers in developing countries can be used by relatively untrained persons to quickly determine the cyanogenic potential of cassava flour (*10*). Wholesalers and government food inspectors can use this method to routinely measure cyanogenic content of commercial cassava flour. Farmers and consumers in areas that depend upon cassava should be warned about cyanide poisoning caused by eating improperly processed or wild-cultivar cassava, and instructed to strictly adhere to the established processing methods to degrade cyanogenic glycosides.

**Summary**

**What is already known about this topic?**

Cassava, an edible tuberous root often made into flour, contains cyanogenic glycosides, which can result in fatal cyanide poisoning if not properly detoxified by soaking, drying, and scraping before being consumed. Acute cassava-associated cyanide poisoning outbreaks are rarely described.

**What is added by this report?**

In September 2017, an outbreak of suspected cyanide poisoning, involving 98 cases with two deaths, occurred in western Uganda. Epidemiologic and laboratory investigation identified consumption of a cassava flour dish made from wild cultivars of cassava with high cyanogenic content as the cause of the outbreak.

**What are the implications for public health practice?**

Education of farmers and consumers about the importance of strict adherence to established methods of degrading cyanogenic glycosides in cassava is essential to prevent cyanide poisoning.
